# Multisystem inflammatory syndrome drug treatment in countries with different income profiles: a scoping review

**DOI:** 10.3389/fphar.2023.1228986

**Published:** 2023-08-23

**Authors:** Luis Phillipe Nagem Lopes, Lidiane Gomes da Cunha, Alice Ramos Oliveira Silva, Marcelo Gerardin Poirot Land, Adriana Rodrigues Fonseca, Luciane Cruz Lopes, Elisangela Costa Lima

**Affiliations:** ^1^ Post-Graduate Program in Pharmaceutical Sciences, University of Sorocaba, São Paulo, Brazil; ^2^ Faculty of Pharmacy, Federal University of Rio de Janeiro, Rio de Janeiro, Brazil; ^3^ Post-Graduate Program in Saúde Materno Infantil, Federal University of Rio de Janeiro, Rio de Janeiro, Brazil

**Keywords:** multisystem inflammatory syndrome in children, social determinants of health, COVID-19, high-cost medicines, pediatrics

## Abstract

**Objective:** The purpose of this study was to map and describe the studies that have investigated therapeutic alternatives for the management of paediatric multisystem inflammatory syndrome in children (MIS-C) associated with COVID-19. Considering the origin of the studies performed (low-, middle- and high-income countries), a systematic scoping review was conducted with primary studies that reported the use of medications for the treatment of patients with MIS-C.

**Sources:** The searches were performed in MEDLINE, Embase, Lilacs, Epistemonikos, CINAHL, and CENTRAL, in the grey literature (theses and dissertations from CAPES, ProQuest, and PROSPERO) and in clinical trial databases until May 2022. The selection and extraction of studies were performed independently by two reviewers.

**Summary of the findings:** A total of 173 studies were included, most of which were published as case reports or series. No randomized controlled clinical trials (RCTs) were identified. The investigated drugs were immunoglobulins, glucocorticoids, monoclonal antibodies, anticoagulants, and antiplatelet agents.

**Conclusion:** The dosages, when reported, were heterogeneous among the studies. The ethnicity and comorbidity of the participants were poorly reported. Monoclonal antibodies, drugs with higher costs, were mostly described in studies of high-income countries.

## Introduction

Multisystem inflammatory syndrome in children (MIS-C) associated with COVID-19 is considered a new postviral hyperinflammatory condition that develops between four and 6 weeks after infection by SARS-CoV-2. It affects children and adolescents and presents fever as a universal symptom followed by multisystem involvement, including cardiovascular complications (Kabeerdoss et al., 2021; Takia et al., 2021).

Since April 2020, MIS-C reports have expanded worldwide, and there has been variation in its definition and treatment (Kabeerdoss et al., 2021; Takia et al., 2021). As of December 2022, 9,333 cases of MIS-C had been reported and caused 76 deaths (0.81%) in the United States (Centers for Disease Control and Prevention, 2023a). In Brazil, 1,970 cases and 135 deaths (6.8%) were reported during the same period. There are indications that MIS-C is more severe in Latin America, given the death rate (Antúnez-Montes et al., 2020).

Although it is considered a rare syndrome, MIS-C should be treated as a public health emergency and may require intensive care and constant surveillance in areas with a high burden of COVID-19 and consistent patterns of racial, socioeconomic, and ethnic differences (Asghar et al., 2022). MIS-C has been less frequently diagnosed in low- and middle-income countries, but it has had a higher proportion of deaths in these places (Asghar et al., 2022). These findings may be related to underdiagnosis, underreporting, or access to therapeutic options. Previous studies have found that racial, ethnic, and socioeconomic disparities were significant barriers to providing specialized health facilities, vaccines, and drugs (Kiss et al., 2021).

Despite numerous publications on the disease, studies that evaluate treatment strategies are scarce (Asghar et al., 2022). In paediatric populations, the evidence is inconsistent due to the scarcity of public health data worldwide (Hoste et al., 2021). Nevertheless, the World Health Organization (WHO) has issued a recommendation on the use of human intravenous immunoglobulin (IVIG) and glucocorticoids, especially methylprednisolone, based on observational studies (World Health Organization, 2021). Other studies reported the use of therapies with monoclonal antibodies, nonsteroidal anti-inflammatory drugs, and anticoagulants for selected cases (Wang et al., 2022). However, in low- and middle-income countries, immunomodulatory therapies are less accessible due to the high cost of these drugs (Jiang et al., 2020; Wang et al., 2022). Socioeconomic differences between countries may have influenced the performance of studies related to pharmacotherapeutic options for the care of patients with MIS-C (Asghar et al., 2022).

The literature on disparities in the COVID-19 context has largely addressed the adult population, while the extent of racial, socioeconomic, and ethnic disparities regarding COVID-19 in children is relatively unknown, especially regarding interventions for the pharmacological management of MIS-C (Asghar et al., 2022).

This scoping review aimed to map the evidence that investigated pharmacological interventions used for treating MIS-C associated with COVID-19 and to characterize the profile, study origin (high-, middle- and low-income countries), drugs, and doses reported.

## Methods

### Study design, protocol and registry

We followed the methodological guidelines of the Joanna Briggs Institute (Peters et al., 2020) and was reported according to the Preferred Reporting Items for Systematic reviews and Meta-Analyses extension for Scoping Reviews (PRISMA-ScR) (Supplementary Material S1). The protocol of this scoping review was previously developed and is available in the Open Science Framework (OSF) (Lopes et al., 2023).

### Eligibility criteria

#### Population

Studies that included neonates, children, and adolescents (0–18 years old) with a confirmed diagnosis of MIS-C associated with COVID-19 based on validated diagnostic criterion were included (Kiss et al., 2021).

#### Outcome/outcome

The outcomes of interest in this review were pharmacological interventions related to the management of MIS-C.

#### Types of studies

Observational or experimental clinical studies were included. Observational studies were included even if they did not measure any efficacy or safety outcome of pharmacological interventions.

#### Sources of information

The following databases were searched until May 2022: MEDLINE (via PubMed), Embase, Lilacs, Epistemonikos, CINAHL, and Cochrane Central Register of Controlled Trials (CENTRAL).

#### Other information resources

1. The grey literature searched included catalogues of theses and dissertations from CAPES, ProQuest, and PROSPERO and repositories of clinical trials registries such as Clinical Trials, WHO International Clinical Trials, Registry Platform Current Controlled Trials and EU Clinical Trials Register.
2. Hand searches were conducted in scientific databases and proceedings of specialized conferences on paediatrics and infectious diseases (Supplementary Material S2).
3. Automatic alerts from scientific databases were generated to identify studies that were published until March 20, 2022.
4. The reference list of the included studies was checked to identify potentially eligible studies. In addition, secondary studies, such as systematic reviews and scoping reviews, were also checked.

#### Search strategies

The keywords were chosen according to the Medical Subject Headings (MeSH) terms to identify relevant studies. The search strategy was developed and validated by a librarian. Subsequently, the search strategy was adapted for the databases and other information sources (Supplementary Material S3). The complete search in each database is described in OSF (Lopes et al., 2023).

#### Determination of eligibility

References were managed in Mendeley software, and duplicates were automatically removed. The abstracts and titles were evaluated by pairs of reviewers (LPNL and AROS and TLA and LGC) independently to verify if they met the eligibility criteria based on Rayyan (Ouzzani et al., 2016). A complete reading of the article was performed by the same pairs of reviewers (LPNL and AROS and TLA and LGC) independently to confirm the eligibility of the studies. Discrepancies were resolved by consensus or by a third reviewer (RCT). The reviewers underwent a calibration process prior to determining eligibility.

#### Data extraction

The information was organized in a Microsoft Excel spreadsheet; the same reviewers independently extracted the data (LPNL and AROS). Discrepancies were resolved by consensus or by a third reviewer (ECL). Previously, the reviewers were calibrated by extracting at least three documents of different levels of complexity, and divergences were discussed. For this study, the following data were considered: 1) Study characteristics: country, study design, and bibliometric information; 2) Patient characteristics: age group, MIS-C diagnostic criteria, comorbidities, and ethnicity; and 3) Characteristics of pharmacological interventions: pharmacological class, drug name, dosage and duration of treatment.

#### Summary of results

For the synthesis of the results, the guidelines of the Synthesis without meta-analysis (SWiM) were followed (Rodgers et al., 2009; Campbell et al., 2020). The data were organized and categorized according to the pharmacological interventions described in the study. Descriptive analyses such as absolute and relative frequencies were performed.

Additionally, the investigated drugs were categorized and compared between low- and middle-income countries *versus* high-income countries. Low-income countries were defined as those with *per capita* income of US$1,045 or less, while high- and middle-income countries were defined as those with *per capita* income between US$4,096 and US$12,695 (The World Bank, 2021).

#### Statistical analysis

Data were presented in numbers (percentages) for categorical variables, and differences between groups were assessed by Pearson chi-square or Fisher’s exact test. For continuous variables data were shown as mean ± standard deviation for normal distribution data and median (Interquartile range—IQR) for non-normal distribution. Chi-square and Student’s t tests were used to compare the profile of scientific evidence on the pharmacological management of MIS-C in low- and middle-income countries *versus* high-income countries. A value of *p* < 0.05 was adopted as statistically significant.

#### Open science

The database containing all the information from the included studies used in the analyses is available in open access (Lopes et al., 2023).

## Results

### Selection of studies

**FIGURE 1:**
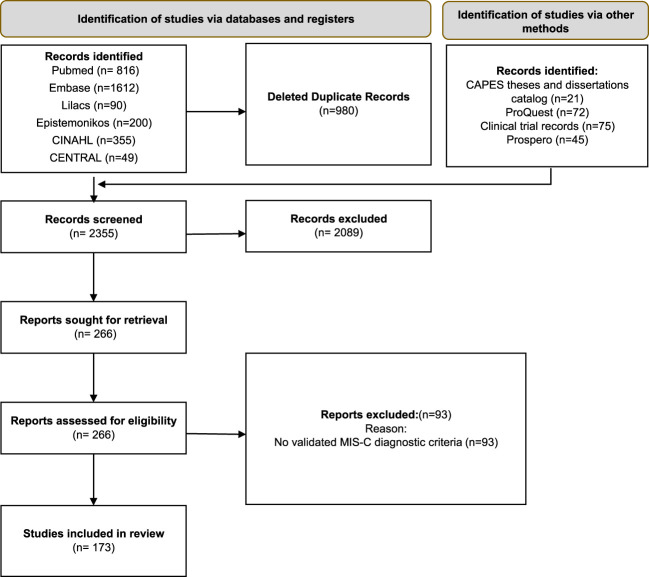
Flowchart of the selection process (PRISMA).

The search in scientific databases resulted in 3122 records. After removal of duplicates (n = 980), 2355 studies were subjected to screening of titles and abstracts. Of these, 2089 records were excluded. The full texts of 266 studies were analysed, of which 173 met the eligibility criteria and 93 were excluded, because they did not have validated diagnostic criteria for MIS-C. The reasons for exclusion after full-text screening are presented in the Supplementary Material S4. Figure 1 shows the PRISMA flowchart referring to the study selection process.

### Characterization of the included studies

**FIGURE 2:**
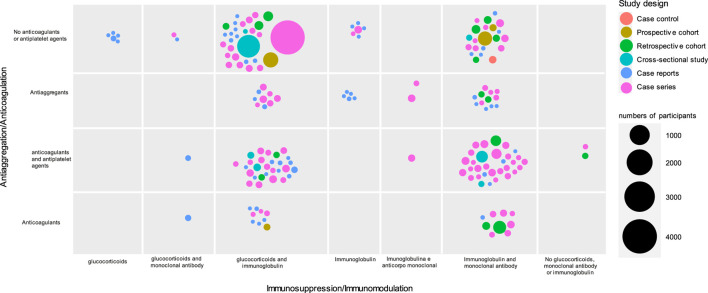
Distribution of included studies according to number of participants, design and reported treatment (n = 173).

All included studies (n = 173) had observational designs, and these were mainly case reports (n = 59, 34.10%) or case series (n = 87, 50.30%) (Figure 2). Most studies included fewer than 100 children (mean = 69.7; median = 13). A study of Centers for Disease Control (CDC) Team reported more than 4,000 cases using a standardized case report form. No randomized controlled clinical trials (RCTs) were identified in the search period of this systematic scoping review. In ongoing RCT databases, only four were identified that addressed pharmacological interventions. Of these, one was published as a protocol (Welzel et al., 2022), and the others did not present results until 2022.

The studies mostly used the CDC (n = 76, 44.00%) and WHO (n = 55, 31.80%) criteria for the MIS-C diagnosis. Some studies (n = 12, 6.90%) used both criteria for the inclusion of participants.

Regarding the clinical-epidemiological characteristics of the participants, all studies reported inflammatory markers, but few studies reported the ethnicity (n = 59, 34.10%) and comorbidities (n = 66, 38.20%). The most described pharmacological treatment was IVIG immunomodulator (n = 162, 93.60%), glucocorticoids (n = 157, 90.80%), or a combination of both (n = 147, 85%). For studies described. Only four studies (2.31%) described the use of steroids alone.

More than half of the studies (n = 93, 53.80%) reported the use of monoclonal antibodies (above all anakinra, tocilizumab, eculizumab, rituximab, infliximab) and nonsteroidal anti-inflammatory drugs (n = 91, 52.60%). In part of the studies, the use of monoclonal antibodies was justified by the intensification or lack of response to the treatment initially used.

The use of anticoagulants (n = 82, 47.40%) was also described in some analyses as management for MIS-C (Table 1).

**TABLE 1:** Characteristics of the included studies (n = 173).

Characteristics	High-income countries	Low and middle-income countries	*p*-value
Year of publication			
2020	33 (30.30)	10 (15.60)	0.092
2021	63 (57.80)	46 (71.90)	
2022	13 (11.90)	8 (12.50)	
Study design			
Case series	55 (50.50)	32 (50.00)	0.620
Case reports	34 (31.20)	25 (39.10)	
Retrospective cohort	12 (11.00)	3 (4.70)	
Cross sectional	4 (3.7)	3 (4.7)	
Prospective cohort	3 (2.80)	1 (1.60)	
Case control	1 (0.90)	0 (0.00)	
Diagnosis of MIS-C according to validated criteria			
CDC	51 (46.80)	25 (39.10)	0.162
WHO	27 (24.80)	28 (43.80)	
RCPCH	19 (17.40)	6 (9.40)	
CDC and WHO	8 (7.30)	4 (6.20)	
Others	4 (3.70)	1 (1.50)	
Clinical and epidemiological characteristics present			
Ethnicity	53 (48.60)	6 (9.40)	<0.001
Comorbidity	45 (41.30)	21 (32.80)	0.344
Inflammatory markers	109 (100)	64 (100)	-
Dose reporting	74 (67.90)	45 (70.30)	0.871
Pharmacological treatment described			
IVIG	100 (91.70)	61 (95.30)	0.560
Glucocorticoids	95 (87.20)	61 (95.30)	0.140
IVIG + Glucocorticoids	89 (81.70)	58 (90.60)	0.169
Monoclonal antibodies	65 (59.60)	15 (23.40)	<0.001
Antiplatelet agents/Anti-inflammatory drugs	56 (51.40)	35 (54.70)	0.792
Systemic anticoagulants	49 (45.00)	33 (51.60)	0.495
Total	109	64	-

### Country of origin of the studies

**FIGURE 3:**
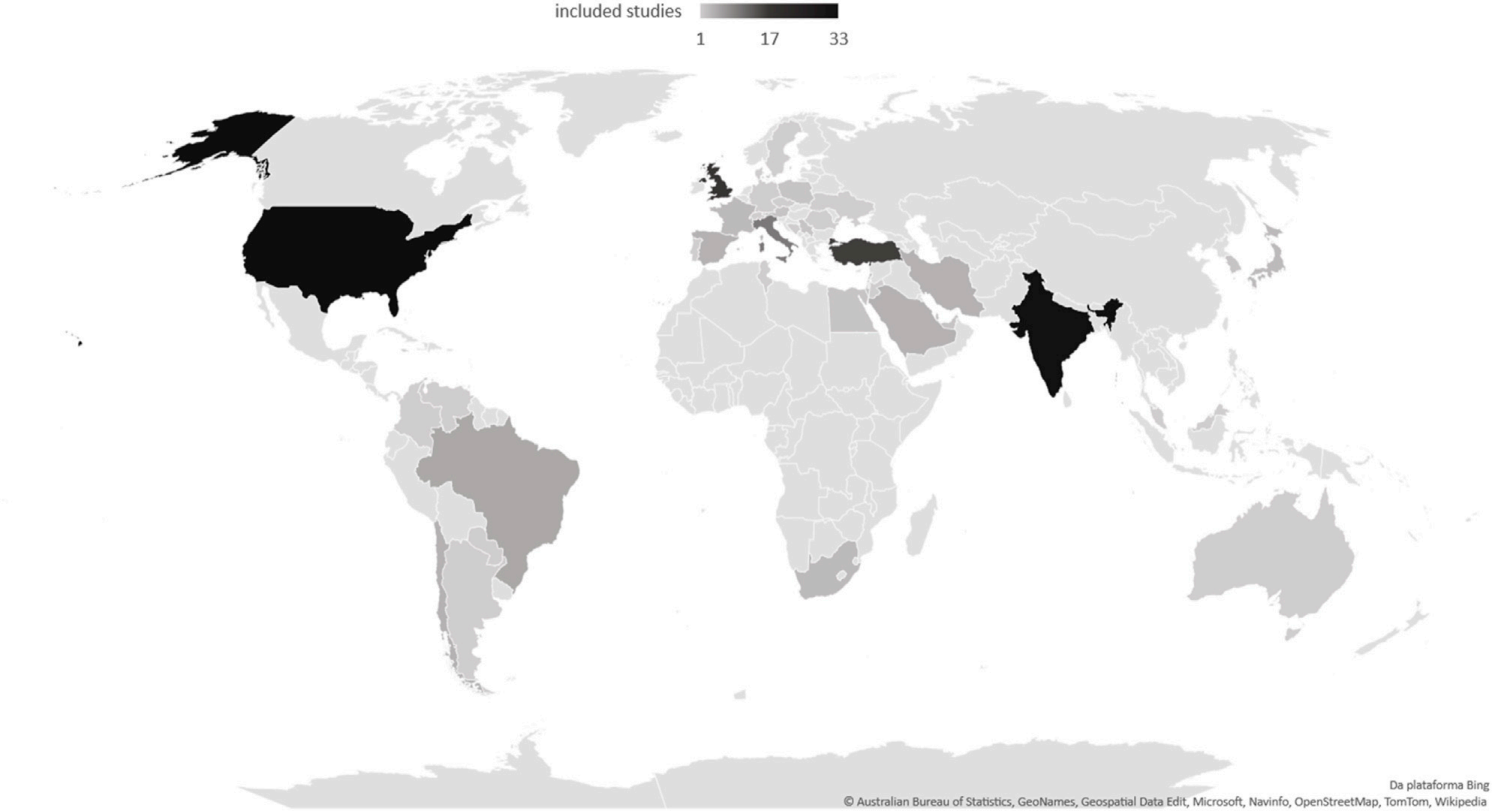
Geographic distribution of studies (Number of studies: 173).

In the case of low- and middle-income countries, a smaller volume of studies and a significant difference in the description of the use of monoclonal antibodies were evident (Table 1). In addition, regarding the characteristics of the studies, high-income countries reported the ethnicity of the participants more often, with statistical significance. Figure 3 illustrates the frequency of studies included in this scoping review.

### Characteristics of the pharmacological interventions described

Of the included studies (n = 173), 119 reported the dosage of at least one pharmacological intervention. Most studies reported the use and dosage of glucocorticoids (n = 107) and IVIG (n = 111). The use and dosage of monoclonal antibodies (63.82%), antiplatelet (63.15%), and anticoagulants (33.52%) were also described. For monoclonal antibodies, the reported dose range was 5–10 mg/kg (infliximab), 4–12 mg/kg (tocilizumab), and 2–400 mg/kg (anakinra). The most frequently reported dosages are described in Table 2.

**TABLE 2:** Dose range described in the included studies.

Medication/Dosage/No.	Dose range analysed in the studies	N (%) of studies	Reported dose variation (minimum - maximum)
Glucocorticoids (mg/kg/day) (n = 107)	Up to 30	83 (77.57)	0.15–1000
	30–1000	24 (22.42)	
	Dose not reported	50 (31.84)	
IGIV (g/kg) (n = 111)	Up to 2	108 (97.29)	0.4–120
	2 to 4	3 (2.70)	
	Dose not reported	51 (31.48)	
Monoclonal antibodies (mg/kg/day) (n = 50)	1 to 10	44 (88.00)	2–400
	Above 10	6 (12.00)	
	Dose not reported	43 (46.23)	
Antiplatelet agents/Anti-inflammatory drugs (mg/kg/day) (n = 61)	3–5 (antiplatelet drugs)	25 (40.98)	3–100
	30–50 (anti-inflammatory)	22 (33.06)	
	Above 50	13 (25.96)	
	Dose not reported	30 (32.96)	
Systemic anticoagulants (mg/kg/day) (n = 58)	≥2 (Therapeutic doses)	15 (25.86)	0.5–120
	Up to 1 (Prophylactic dose)	25 (43.10)	
	Both (therapeutic/prophylactic)	18 (31.04)	
	Dose not reported	24 (29.26)	

## Discussion

### Main findings

As expected, this review indicated that the scientific evidence on the pharmacological management of MIS-C associated with COVID-19 is still insufficient. No RCTs of pharmacological interventions for the treatment of MIS-C were found. Most studies were published as case series and case reports in 2021 and described the use of glucocorticoids and IVIG, alone or in combination. These drugs were recommended by the WHO in November 2021 (World Health Organization, 2021).

The COVID-19 pandemic has generated significant social adaptations at the individual, community, and global levels (Ahmadi et al., 2021). This also affected clinical research (Park et al., 2021). At the time of publication, the WHO clinical guidelines for the management of MIS-C considered three observational studies, which gathered information from 885 patients and were the best evidence available at the time to meet public health demands. There is still a lack of RCTs, which are considered the gold standard for evaluating the safety of health interventions (Pasquali et al., 2012). The studies available and included in this review are characterized by having a high risk of bias (lack of a comparison group) and several confounders (Murad et al., 2018).

The most used diagnostic criteria for MIS-C in this review were those of the WHO and CDC, a finding similar to the systematic review conducted by Hoste et al., 2021; Hoste et al., 2021). In January 2023, the CDC updated the definition criteria for MIS-C, making them even more like those of the WHO (Centers for Disease Control and Prevention, 2023b).

The scarcity of studies in Africa does not reflect the lack of MIS-C cases in this continent (Butters et al., 2022). In addition, the economic profile of a country influences its scientific production (Rodríguez-Navarro and Brito, 2022). In low- and middle-income countries, many physicians working on the front lines during the COVID-19 outbreak were overloaded with patient care, making it difficult to allocate time for clinical research (Asghar et al., 2022). This factor may explain, in part, the lower number of publications in low- and middle-income countries compared to high-income countries, as well as the access to more expensive drugs.

Few studies included in this scoping review reported the ethnicity of the participants, and this difference in reporting was statistically significant in studies conducted by countries with different income profiles. The WHO recommended that policymakers, managers, and health professionals consider health equity in care plans during the COVID-19 pandemic (World Health Organization, 2023). Burt have a 15-fold increased risk of developing ISM-C after SARS-CoV-2 infection, which may be associated with genetic and socioeconomic factors and access to health services by this population (Dong et al., 2020; Javalkar et al., 2021). Thus, the absence of these data makes it challenging to formulate pharmaceutical policies that consider equity in implementing these therapeutic options.

Only 38.20% described associated comorbidities. Asghar et al. (2022) found that the most common comorbidities observed in patients with MIS-C were obesity, asthma, hypothyroidism, non-alcoholic fatty liver disease, respiratory disease preceding 4 weeks of hospitalization, and glucose-6-phosphate dehydrogenase deficiency. However, it is still unclear whether the observed comorbidities required specific treatments in the scenario of MIS-C associated with COVID-19 (World Health Organization, 2021). Comorbidities may also influence the safety and efficacy of pharmacotherapy.

The most often described pharmacological classes were glucocorticoids and IVIG, used separately or in combination. This result was similar for all countries analysed, that is, low-, middle-income and high-income countries. This finding was expected, considering that these are the treatment options recommended by the WHO clinical guidelines and by rheumatological and/or paediatric societies (Harwood et al., 2021; World Health Organization, 2021; Henderson et al., 2022).

Tumor necrosis factor (TNF) inhibitor monoclonal antibodies (infliximab), interleukin (IL) 1 inhibitors (anakinra, canakinumab), and IL-6 inhibitors (tocilizumab) are high-cost drugs described in 53.80% of the studies. These studies were mainly conducted in high-income countries, and a part specified the use for refractory cases. Although the number of studies assessed does not fully represent the total number of patients with MIS-C who received treatment with monoclonal antibodies, because they were observational studies, designs closest to those based on real-world data, it can be inferred that patients in high-income countries had greater access to these drugs. High-cost drugs represent a major challenge to pharmaceutical policies (Organização Pan-Americana Da Saúde, 2003), such as low levels of coverage, financial fragility of health systems, limitations of drug distribution networks, and the general problems of access to health services for a large part of the population (Van Der Ent, 2017). These factors were likely exacerbated by COVID-19, as a global supply chain threat mainly linked to health products, such as medicines.

These drugs are seen as an alternative therapy for the treatment of MIS-C for patients who cannot receive glucocorticoids or in patients unresponsive to previous treatment with IVIG and glucocorticoids at low to moderate doses (Henderson et al., 2022). Two studies reported the use of tocilizumab or anakinra associated with glucocorticoids without human immunoglobulin as initial management in children diagnosed with MIS-C before the publication of WHO recommendations (Calò Carducci et al., 2020; Niño-Taravilla et al., 2020).

The dose of the used drugs was heterogeneous, a result that was also expected, given the challenges of determining therapeutic protocols for children and adolescents, since the growth and development of the organism considerably influence the pharmacokinetics and pharmacodynamics (Batchelor and Marriott, 2015).

Among the studies that reported the doses, similarities were identified when compared to the WHO recommendations (World Health Organization, 2021). Our findings suggest, however, that the posology definition of anticoagulants in MIS-C is still a challenge, a fact observed by the wide dose intervals in the studies. This can be explained by the variation in the ideal dosage observed in the pharmacological protocols of health institutions regarding thromboprophylactic practices in paediatric patients during the SARS-CoV-2 pandemic (Whitworth et al., 2021).

It is noteworthy that care, concerning the risk of a thrombotic event, extends from admission to hospital discharge (Whitworth et al., 2021), where children with MIS-C should be evaluated individually because therapeutic anticoagulation must counterbalance between the patient’s risk factors and possible risk of bleeding during the use of these agents (Whitworth et al., 2021). The main risk factors for thrombosis in patients with ISM-C are i) use of a central venous catheter, ii) admission to the intensive care unit, iii) D-dimer measurement five times above the reference value, iv) age older than 12 years and v) cancer (Whitworth et al., 2021). However, more studies are needed to overcome this gap regarding the use of anticoagulants in children with MIS-C.

Regarding antiplatelet therapy, the studies included in this analysis described the use of dosage ranges compatible with those reported and recommended by international consensus (World Health Organization, 2021; Henderson et al., 2022).

This is the first scoping review to exhaustively map the pharmacological interventions used to manage MIS-C during the COVID-19 pandemic and to consider social determinants, such as the income profile of the countries, in the production of scientific evidence on the pharmacological management of MIS-C. The recommended guidelines for scoping reviews were followed, including a complete scrutiny of the literature, automatic alerts of databases, and study selection and data extraction performed by pairs of reviewers independently (Peters et al., 2020). Experts on the subject were not consulted to identify relevant studies. This limitation was mitigated by checking the references of the included studies and the identified systematic reviews. In addition, the presence of a pharmacist (LGC) and a physician (ARF) with expertise in MIS-C care as authors of this review may have reduced this limitation.

It is also important to consider that secondary studies that discuss the social and economic disparities of countries are generally underestimated (Garlinghouse M, 2023). This is because the acceptance rates of articles are higher when the first authors come from high-income English-speaking countries, and articles from high-income countries have higher citation rates (Smith R, 2017). Although the objective of this scoping review was not to discuss this phenomenon but to raise hypotheses for further studies based on the identified gaps, it is important to consider this trend in publishing (Harris et al., 2017).

Although this study used a rigorous methodological process in its development, no RCTs provided data on the safety and efficacy of pharmacological interventions for managing MIS-C. It is noteworthy that there is an ongoing RCT evaluating the efficacy and safety of methylprednisolone *versus* intravenous immunoglobulins in ISM-C (Welzel et al., 2023). Furthermore, no evaluation of the methodological quality of the included studies was performed, as scoping reviews are designs used mainly for mapping evidence (Peters et al., 2020). Therefore, the assessment of the risk of bias is not mandatory.

### Implications for researchers

Our scoping review did not identify any RCTs. This finding reflects the pandemic and the challenges inherent in developing RCTs with the paediatric population. It is known that data on the safety and efficacy of many drugs used in children are surprisingly scarce (Joseph et al., 2015). As a result, children are often given drugs without proven efficacy or drugs with unknown adverse effects (Lagler et al., 2021; Santos et al., 2022). Thus, RCTs with methodological quality and with significant samples need to be designed to improve knowledge about the safety and efficacy of drugs for the management of MIS-C. In addition, strategies that include real-world evidence need to be considered by researchers when RCTs are not ethically and financially viable.

Current studies lack data on race- and ethnicity-specific presentations of the syndrome, the mechanism of genetic predisposition to MIS-C and its worldwide distribution. Social determinants of health should be routinely considered in clinical assessments in the same way as age and sex, as they can play an important role in helping to create personalized risk mitigation policies. In view of the importance of scientific evidence to inform effective public policies that consider implementation factors related to equity, it is important that researchers are sensitized to consider the report of ethnicity and income in their studies.

### Implications for clinical practice and public policy

The findings of this scoping review indicate that high-cost drugs were more often investigated in high-income countries than in other countries. It is possible that access to these drugs was also greater. In-depth knowledge and use of information about access to health services and technologies in low- and middle-income countries are essential, especially during a global public health emergency, such as the COVID-19 pandemic. This information can lead to elaboration of health policies that consider racial and socioeconomic differences, especially in Brazil.

Health professionals should be aware of the scarcity of safety and efficacy studies on MIS-C treatment. Consideration should be given to the constant monitoring of potential adverse events and the emergence of new evidence that may substantially change pharmaceutical care policies and the decision-making process, since, as this scoping review shows, the evidence is still lacking.

## Conclusion

Most of the studies identified had an observational design. No RCTs were identified. Most pharmacological interventions described were IVIG and glucocorticoids, but monoclonal antibodies, anticoagulants and antiplatelet agents were also described. The doses, when reported, were heterogeneous among the studies. Monoclonal antibodies, high-cost drugs, have been most studied in high-income countries. The publications poorly reported on the ethnicity and comorbidity of the participants.
