# Supplementary material for: Multisystem inflammatory syndrome drug treatment in countries with different income profiles: a scoping review
Source: Front Pharmacol. 2023 Aug 23;14:1228986. doi: 10.3389/fphar.2023.1228986 (PMC10481533; doi:10.3389/fphar.2023.1228986)
Supplement: Supplementary file 2 [file DataSheet1.docx]

Supplementary Material

Multisystem Inflammatory Syndrome (MIS-C) drug treatment in countries with different
income profiles: A scoping review
Luis Phillipe Nagem Lopes^1^#, Lidiane Gomes da Cunha^2^#, Alice Ramos Oliveira Silva^2^, Marcelo Gerardin Poirot Land^3^, Adriana Rodrigues Fonseca³, Luciane Cruz Lopes^4^, Elisangela Costa Lima^2^*
Correspondence: Elisangela Costa Lima: [eclima.ufrj@gmail.com](mailto:eclima.ufrj@gmail.com)

Pubmed (N=816)

1. “multisystem inflammatory syndrome in children” [All Fields]
2. “MIS-C” [All Fields]
3. “PIMS-TS” [All Fields]
4. “pediatric inflammatory multisystem syndrome temporally associated with COVID-19” [All Fields]
5. or/1-4
6. “COVID 19 [All Fields]”
7. “SARS-CoV-2 Infection” [All Fields]
8. “Infection, SARS-CoV-2” [All Fields]
9. “SARS CoV 2 Infection” [All Fields]
10. “SARS-CoV-2 Infections” [All Fields]
11. “2019 Novel Coronavirus Disease” [All Fields]
12. “2019 Novel Coronavirus Infection” [All Fields]
13. “2019-nCoV Disease” [All Fields]
14. “2019 nCoV Disease” [All Fields]
15. “2019-nCoV Diseases” [All Fields]
16. “Disease, 2019-nCoV” [All Fields]
17. “COVID-19 Virus Infection” [All Fields]
18. “COVID 19 Virus Infection” [All Fields]
19. “COVID-19 Virus Infections” [All Fields]
20. “Infection, COVID-19 Virus” [All Fields]
21. “Virus Infection, COVID-19” [All Fields]
22. “Coronavirus Disease 2019” [All Fields]
23. “Disease 2019, Coronavirus” [All Fields]
24. “Coronavirus Disease-19” [All Fields]
25. “Coronavirus Disease 19” [All Fields]
26. “Severe Acute Respiratory Syndrome Coronavirus 2 Infection” [All Fields]
27. “SARS Coronavirus 2 Infection” [All Fields]
28. “COVID-19 Virus Disease” [All Fields]
29. “COVID 19 Virus Disease” [All Fields]
30. “COVID-19 Virus Diseases” [All Fields]
31. “Disease, COVID-19 Virus” [All Fields]
32. “Virus Disease, COVID-19” [All Fields]
33. “2019-nCoV Infection” [All Fields]
34. “2019 nCoV Infection” [All Fields]
35. “2019-nCoV Infections” [All Fields]
36. “Infection, 2019-nCoV” [All Fields]
37. “COVID19” [All Fields]
38. “COVID-19 Pandemic” [All Fields]
39. “COVID 19 Pandemic” [All Fields]
40. “Pandemic, COVID-19” [All Fields]
41. “COVID-19 Pandemics” [All Fields]
42. or/6-42
43. 5 AND 42

Embase (n=1612)

1. ‘multisystem inflammatory syndrome in children’ [All Fields]
2. ‘MIS-C’ [All Fields]
3. ’PIMS-TS’ [All Fields]
4. ‘pediatric inflammatory multisystem syndrome temporally associated with COVID-19’ [All Fields]
5. or/1-4
6. ‘COVID 19 ’ [All Fields]
7. ‘SARS-CoV-2 Infection’ [All Fields]
8. ‘Infection, SARS-CoV-2’ [All Fields]
9. ‘SARS CoV 2 Infection’ [All Fields]
10. ‘SARS-CoV-2 Infections’ [All Fields]
11. ‘2019 Novel Coronavirus Disease’ [All Fields]
12. ‘2019 Novel Coronavirus Infection’ [All Fields]
13. ‘2019-nCoV Disease’ [All Fields]
14. ‘2019 nCoV Disease’ [All Fields]
15. ‘2019-nCoV Diseases’ [All Fields]
16. ‘Disease, 2019-nCoV’ [All Fields]
17. ‘COVID-19 Virus Infection’ [All Fields]
18. ‘COVID 19 Virus Infection’ [All Fields]
19. ‘COVID-19 Virus Infections’ [All Fields]
20. ‘Infection, COVID-19 Virus’ [All Fields]
21. ‘Virus Infection, COVID-19’ [All Fields]
22. ‘Coronavirus Disease 2019’ [All Fields]
23. ‘Disease 2019, Coronavirus’ [All Fields]
24. ‘Coronavirus Disease-19’ [All Fields]
25. ‘Coronavirus Disease 19’ [All Fields]
26. ‘Severe Acute Respiratory Syndrome Coronavirus 2 Infection’ [All Fields]
27. ‘SARS Coronavirus 2 Infection’ [All Fields]
28. ‘COVID-19 Virus Disease’ [All Fields]
29. ‘COVID 19 Virus Disease’ [All Fields]
30. ‘COVID-19 Virus Diseases’ [All Fields]
31. ‘Disease, COVID-19 Virus’ [All Fields]
32. ‘Virus Disease, COVID-19’ [All Fields]
33. ‘2019-nCoV Infection’ [All Fields]
34. ‘2019 nCoV Infection’ [All Fields]
35. ‘2019-nCoV Infections’ [All Fields]
36. ‘Infection, 2019-nCoV’ [All Fields]
37. COVID19 [All Fields]
38. ‘COVID-19 Pandemic’[All Fields]
39. ‘COVID 19 Pandemic’ [All Fields]
40. ‘Pandemic, COVID-19’ [All Fields]
41. ‘COVID-19 Pandemics’ [All Fields]
42. or/6-42
43. 5 AND 42

LILACS (Via BVS) (N=90)

1. “multisystem inflammatory syndrome in children” [All Fields]
2. “MIS-C” [All Fields]
3. “PIMS-TS” [All Fields]
4. “pediatric inflammatory multisystem syndrome temporally associated with COVID-19” [All Fields]
5. “Síndrome Inflamatória Multissistêmica em Crianças” [All Fields]
6. “Síndrome Inflamatória Multissistêmica Pediatrica Temporariamente associada ao SARS-CoV-2” [All Fields]
7. “Síndrome Inflamatória Multissistêmica Pediatrica Temporariamente associada a COVID-19” [All Fields]
8. “Síndrome Inflamatória Multissistêmica Pediatrica” [All Fields]
9. or/1-8
10. “COVID 19” [All Fields]
11. “SARS-CoV-2 Infection” [All Fields]
12. “Infection, SARS-CoV-2” [All Fields]
13. “SARS CoV 2 Infection” [All Fields]
14. “SARS-CoV-2 Infections” [All Fields]
15. “2019 Novel Coronavirus Disease” [All Fields]
16. “2019 Novel Coronavirus Infection” [All Fields]
17. “2019-nCoV Disease” [All Fields]
18. “2019 nCoV Disease” [All Fields]
19. “2019-nCoV Diseases” [All Fields]
20. “Disease, 2019-nCoV” [All Fields]
21. “COVID-19 Virus Infection” [All Fields]
22. “COVID 19 Virus Infection” [All Fields]
23. “COVID-19 Virus Infections” [All Fields]
24. “Infection, COVID-19 Virus” [All Fields]
25. “Virus Infection, COVID-19” [All Fields]
26. “Coronavirus Disease 2019” [All Fields]
27. “Disease 2019, Coronavirus” [All Fields]
28. “Coronavirus Disease-19” [All Fields]
29. “Coronavirus Disease 19” [All Fields]
30. “Severe Acute Respiratory Syndrome Coronavirus 2 Infection” [All Fields]
31. “SARS Coronavirus 2 Infection” [All Fields]
32. “COVID-19 Virus Disease” [All Fields]
33. “COVID 19 Virus Disease” [All Fields]
34. “COVID-19 Virus Diseases” [All Fields]
35. “Disease, COVID-19 Virus” [All Fields]
36. “Virus Disease, COVID-19” [All Fields]
37. “2019-nCoV Infection” [All Fields]
38. “2019 nCoV Infection” [All Fields]
39. “2019-nCoV Infections” [All Fields]
40. “Infection, 2019-nCoV” [All Fields]
41. “COVID19” [All Fields]
42. “COVID-19 Pandemic” [All Fields]
43. “COVID 19 Pandemic” [All Fields]
44. “Pandemic, COVID-19” [All Fields]
45. “COVID-19 Pandemics” [All Fields]
46. or/10-45
47. 9 AND 46

Epistemonikos (N=200)

1. “multisystem inflammatory syndrome in children” [All Fields]
2. “MIS-C” [All Fields]
3. “PIMS-TS” [All Fields]
4. “pediatric inflammatory multisystem syndrome temporally associated with COVID-19” [All Fields]
5. or/1-4
6. “COVID 19” [All Fields]
7. “SARS-CoV-2 Infection” [All Fields]
8. “Infection, SARS-CoV-2” [All Fields]
9. “SARS CoV 2 Infection” [All Fields]
10. “SARS-CoV-2 Infections” [All Fields]
11. “2019 Novel Coronavirus Disease” [All Fields]
12. “2019 Novel Coronavirus Infection” [All Fields]
13. “2019-nCoV Disease” [All Fields]
14. “2019 nCoV Disease” [All Fields]
15. “2019-nCoV Diseases” [All Fields]
16. “Disease, 2019-nCoV” [All Fields]
17. “COVID-19 Virus Infection” [All Fields]
18. “COVID 19 Virus Infection” [All Fields]
19. “COVID-19 Virus Infections” [All Fields]
20. “Infection, COVID-19 Virus” [All Fields]
21. “Virus Infection, COVID-19” [All Fields]
22. “Coronavirus Disease 2019” [All Fields]
23. “Disease 2019, Coronavirus” [All Fields]
24. “Coronavirus Disease-19” [All Fields]
25. “Coronavirus Disease 19” [All Fields]
26. “Severe Acute Respiratory Syndrome Coronavirus 2 Infection” [All Fields]
27. “SARS Coronavirus 2 Infection” [All Fields]
28. “COVID-19 Virus Disease” [All Fields]
29. “COVID 19 Virus Disease” [All Fields]
30. “COVID-19 Virus Diseases” [All Fields]
31. “Disease, COVID-19 Virus” [All Fields]
32. “Virus Disease, COVID-19” [All Fields]
33. “2019-nCoV Infection” [All Fields]
34. “2019 nCoV Infection” [All Fields]
35. “2019-nCoV Infections” [All Fields]
36. “Infection, 2019-nCoV” [All Fields]
37. “COVID19” [All Fields]
38. “COVID-19 Pandemic” [All Fields]
39. “COVID 19 Pandemic” [All Fields]
40. “Pandemic, COVID-19” [All Fields]
41. “COVID-19 Pandemics” [All Fields]
42. or/6-42
43. 5 AND 42

CINAHL (N=355)

1. “multisystem inflammatory syndrome in children” [All Fields]
2. “MIS-C” [All Fields]
3. “PIMS-TS” [All Fields]
4. “pediatric inflammatory multisystem syndrome temporally associated with COVID-19” [All Fields]
5. or/1-4
6. “COVID 19” [All Fields]
7. “SARS-CoV-2 Infection” [All Fields]
8. “Infection, SARS-CoV-2” [All Fields]
9. “SARS CoV 2 Infection” [All Fields]
10. “SARS-CoV-2 Infections” [All Fields]
11. “2019 Novel Coronavirus Disease” [All Fields]
12. “2019 Novel Coronavirus Infection” [All Fields]
13. “2019-nCoV Disease” [All Fields]
14. “2019 nCoV Disease” [All Fields]
15. “2019-nCoV Diseases” [All Fields]
16. “Disease, 2019-nCoV” [All Fields]
17. “COVID-19 Virus Infection” [All Fields]
18. “COVID 19 Virus Infection” [All Fields]
19. “COVID-19 Virus Infections” [All Fields]
20. “Infection, COVID-19 Virus” [All Fields]
21. “Virus Infection, COVID-19” [All Fields]
22. “Coronavirus Disease 2019” [All Fields]
23. “Disease 2019, Coronavirus” [All Fields]
24. “Coronavirus Disease-19” [All Fields]
25. “Coronavirus Disease 19” [All Fields]
26. “Severe Acute Respiratory Syndrome Coronavirus 2 Infection” [All Fields]
27. “SARS Coronavirus 2 Infection” [All Fields]
28. “COVID-19 Virus Disease” [All Fields]
29. “COVID 19 Virus Disease” [All Fields]
30. “COVID-19 Virus Diseases” [All Fields]
31. “Disease, COVID-19 Virus” [All Fields]
32. “Virus Disease, COVID-19” [All Fields]
33. “2019-nCoV Infection” [All Fields]
34. “2019 nCoV Infection” [All Fields]
35. “2019-nCoV Infections” [All Fields]
36. “Infection, 2019-nCoV” [All Fields]
37. “COVID19” [All Fields]
38. “COVID-19 Pandemic” [All Fields]
39. “COVID 19 Pandemic” [All Fields]
40. “Pandemic, COVID-19” [All Fields]
41. “COVID-19 Pandemics” [All Fields]
42. or/6-42
43. 5 AND 42

Cochrane (n=49)

1. COVID-19 [All Fields]
2. SARS-COV-2 [All Fields]
3. or/1-2
4. “multisystem inflammatory syndrome in children” [All Fields]
5. 3 AND 4

Literatura cinzenta nacional: Teses e dissertações Portal CAPES e BDTDs

1. “Síndrome Inflamatória Multissistêmica Pediatrica Temporariamente associada ao SARS-CoV-2” [All Fields]
2. “Síndrome Inflamatória Multissistêmica Pediatrica Temporariamente associada a COVID-19” [All Fields]
3. “Síndrome Inflamatória Multissistêmica Pediatrica” [All Fields]
4. or/1-3
5. COVID 19 [All Fields]
6. SARS-COV-2 [All Fields]
7. Or/5-6
8. 4 AND 7

Literatura cinzenta internacional:Prospero (n=45) e ProQuest (n=72)

1. COVID-19 [All Fields]
2. SARS-COV-2 [All Fields]
3. or/1-2
4. “multisystem inflammatory syndrome in children” [All Fields]
5. 3 AND 4

Literatura específica de registro de ensaios clínicos: Clinical Trials (n=22), WHO International Clinical Trials (=18), Registry Platform Current Controlled Trials (n=25) e EU Clinical Trials Register (n=10)

1. COVID-19 [All Fields]
2. SARS-COV-2 [All Fields]
3. or/1-2
4. “multisystem inflammatory syndrome in children” [All Fields]
5. 3 AND 4
